# Greater rate of return to play and re-injury following all-inside meniscal repair compared to the inside-out technique: a systematic review

**DOI:** 10.1007/s00402-023-04933-8

**Published:** 2023-06-07

**Authors:** Filippo Migliorini, Giovanni Asparago, Francesco Oliva, Andreas Bell, Frank Hildebrand, Nicola Maffulli

**Affiliations:** 1 Department of Orthopaedic, Trauma, and Reconstructive Surgery, RWTH University Hospital, Pauwelsstraße 30, 52074 Aachen, Germany; 2 Department of Orthopedics and Trauma Surgery, Academic Hospital of Bolzano (SABES-ASDAA), 39100 Bolzano, Italy; 3 Department of Medicine, Surgery and Dentistry, University of Salerno, 84081 Baronissi, SA Italy; 4 School of Pharmacy and Bioengineering, Faculty of Medicine, Keele University, ST4 7QB Stoke On Trent, England; 5 Barts and the London School of Medicine and Dentistry, Centre for Sports and Exercise Medicine, Mile End Hospital, Queen Mary University of London, E1 4DG London, England; 6 Department of Orthopaedic and Trauma Surgery, Eifelklinik St. Brigida, Simmerath, Germany

**Keywords:** Meniscal repair, All-inside, Inside-out, Re-injury, Return to sport

## Abstract

**Introduction:**

Inside-out and all-inside arthroscopic meniscal repairs are widely performed. However, it remains unclear which method promotes greater clinical outcomes. This study compared inside-out versus all-inside arthroscopic meniscal repair in terms of patient-reported outcome measures (PROMs), failures, return to play, and symptoms.

**Methods:**

This systematic review was conducted in accordance with the PRISMA guidelines. Two authors independently performed the literature search by accessing the following databases: PubMed, Google Scholar, and Scopus in February 2023. All clinical studies which investigated the outcomes of all-inside and/or inside-out meniscal repair were considered.

**Results:**

Data from 39 studies (1848 patients) were retrieved. The mean follow-up was 36.8 (9 to 120) months. The mean age of the patients was 25.8 ± 7.9 years. 28% (521 of 1848 patients) were women. No difference was found in PROMs: Tegner Activity Scale (*P* = 0.4), Lysholm score (*P* = 0.2), and International Knee Document Committee score (*P* = 0.4) among patients undergoing meniscal repair with all inside or inside-out techniques. All-inside repairs showed a greater rate of re-injury (P = 0.009) but also a greater rate of return to play at the pre-injury level (*P* = 0.0001). No difference was found in failures (*P* = 0.7), chronic pain (*P* = 0.05), reoperation (*P* = 0.1) between the two techniques. No difference was found in the rate of return to play (*P* = 0.5) and to daily activities (*P* = 0.1) between the two techniques.

**Conclusion:**

Arthroscopic all-inside meniscal repair may be of special interest in patients with a particular interest in a fast return to sport, while, for less demanding patients, the inside-out suture technique may be recommended. High-quality comparative trials are required to validate these results in a clinical setting.

**Level of Evidence:**

Level III, systematic review.

## Introduction

The meniscus, a fibrocartilaginous structure essential for stabilizing the knee joint, absorbing shocks, distributes forces and protects the articular cartilage [1–5]. Acute tears of the meniscus may be symptomatic, impacting negatively quality of life and sport participation, and may lead to early onset osteoarthritis [6–9]. Rotational and shear forces on the menisci, especially during kneeling, carrying heavy loads and movements with acceleration, deceleration, jumping, and change of direction, are the main causes of acute tears of the meniscus [10–13]. Direct traumas to the knee might also cause meniscal damage and are often associated with damage to adjacent bone and ligaments [14, 15]. In adults with meniscal degeneration, meniscal tears develop from relatively minor forces or trauma [16, 17].

Meniscal repair is associated with reduced chondral damage compared to meniscectomy [18–22]. In this context, by stabilising the knee joint, meniscal repair prevents cartilage damage, thus preventing early-onset osteoarthritis [23–25]. Repair of the damaged meniscal tissue was introduced in the 1980s [26, 27]. Arthroscopic repair of meniscal injuries has become popular [28–30]. Inside-out and all-inside are two well-established methodologies to repair the damaged meniscus during arthroscopy. Though these techniques are widely performed and validated in several clinical settings, it remains unclear which method promotes greater clinical outcomes. This study compared inside-out versus all-inside arthroscopic meniscal repair in terms of patient-reported outcome measures (PROMs), failures, return to play, and symptoms.

## Methods

### Search strategy

This systematic review was conducted in accordance with the Preferred Reporting Items for Systematic Reviews and Meta-Analyses (PRISMA) [31]. The PICO algorithm was established:

- P (population): meniscal tears in the active population;
- I (intervention): arthroscopic meniscal repair;
- C (comparison): All-inside, inside-out;
- O (outcomes): PROMs, clinical examination, complications.

### Literature search

Two authors (**;**) independently performed the literature search by accessing the following database PubMed, Google Scholar, Scopus in February 2023. The following keywords were used for the search in combination using the Boolean operator AND/OR: meniscal, injury, trauma, acute, defects, tear, rupture, sport, arthroscopy, repair, refixation, all-inside, inside-out. If the title matched the topic, the abstract was read and the full text of the article was accessed. The bibliographies of the articles of interest were screened by hand. Disagreements between the authors were debated and solved by a third author (**).

### Eligibility criteria

All clinical studies which investigated the outcomes of all-inside and/or inside-out meniscal repairs were considered. Articles with levels of evidence I to III, according to the Oxford Centre of Evidenced-Based Medicine [32], were considered. Given the authors language capabilities, articles in English, Italian, French, Spanish, and German were considered. Technical notes, editorials, protocols, comments, guidelines, and reviews were excluded. Biomechanical, animal, and cadaveric studies were also not eligible. Studies that reported data on meniscal procedures augmented with mesenchymal stem cells were not considered. Only articles reporting quantitative data under the outcomes of interest were included.

### Data extraction

Two authors (**,**) independently performed data extraction and collection. The generalities of the studies were retrieved. The length of follow-up, sample size, and percentage of women in each study were collected. The outcomes of interest were the average age of patients at the time of injury, the incidence between male and female sex, the type of meniscal lesion, and the degree of effectiveness of each technique based on the percentage of patients who returned to play and of re-injured. Specifically, the rate of return to play was also assessed, also considering the patients who managed to return to play at a pre-injury level. The following PROMs were evaluated: International Knee Documentation Committee (IKDC) [33], Lysholm Knee Scoring Scale [34], Tegner Activity Scale [35]. Data on the following complication were collected: rate of re-injury, failures, chronic pain, and reoperation. Data concerning the rate of return to play at a pre-injury level, return to play and daily activities were also retrieved.

### Methodological quality assessment

For the methodological quality assessment, the Coleman Methodology Score (CMS) was used [36]. The CMS is a reliable tool to evaluate the methodological quality of systematic reviews and meta-analyses. This score analyses each included study with several endpoints: study size, follow-up duration, surgical approach, type of study, description of the diagnosis, surgical technique, and rehabilitation. The procedures for assessing outcomes and the subject selection process are also evaluated. The CMS rates articles with values between 0 (poor) and 100 (excellent). Articles with values greater than 60 are considered satisfactory.

### Statistical analysis

The statistical analyses were conducted by the first author (**) using the IBM SPSS software (version 25). For descriptive statistics, mean and standard deviation were evaluated. The t-test was performed to assess baseline comparability, with values of *P* > 0.05 considered satisfactory. For the comparisons of continuous data, the mean difference (MD) effect measure and the unpaired t-test were performed. For binary data, the Odd Ratio (OR) effect measure was evaluated. The confidence interval (CI) was set at 0.95. Values of P < 0.05 were considered statistically significant.

## Results

### Study selection

**Fig. 1:**
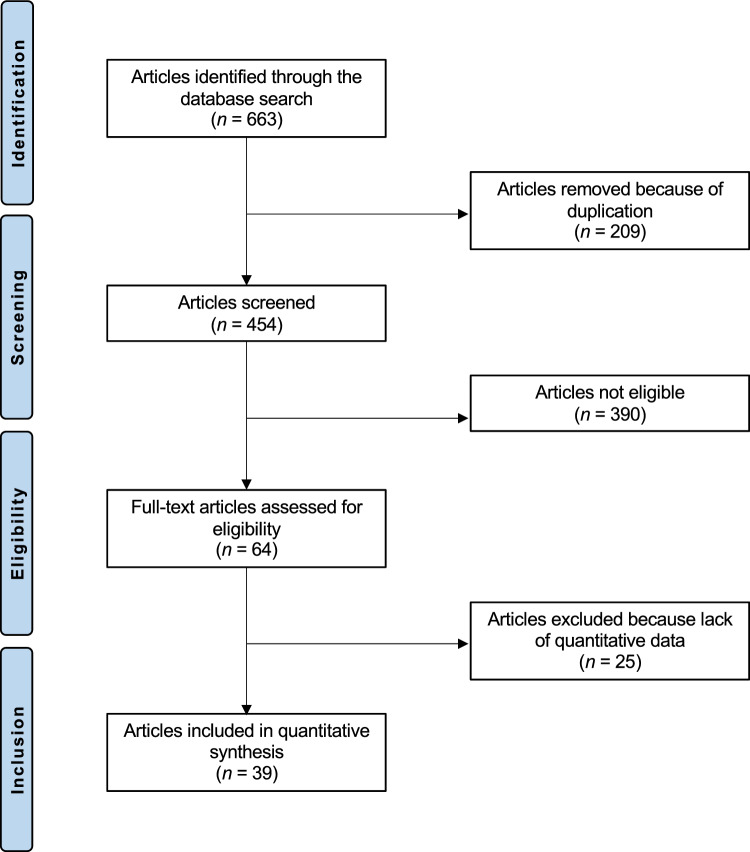
Flow chart of the literature search

The initial literature search resulted in 12,843 articles. Of them, only 663 articles matched the topic. Duplicate records (*N* = 209) were excluded. Of these, a further 390 articles were excluded for reason: not matching the topic (*N* = 271), study design inappropriate (*N* = 112), language limitation (*N* = 4), not available full-text (*N* = 3). A further 25 articles did not report quantitative data under the outcome of interest, and thus, excluded. This left 39 articles for inclusion. Of them, 22 are prospective and 17 are retrospective studies. The literature search results are shown in Fig. 1.

### Study risk of bias assessment

The CMS identified some limitations and strengths in the present study. The size of the study and the duration of follow-up of the included articles were acceptable. The surgical approach, diagnosis and rehabilitation were well described in most of the articles. The outcome measures and timing of the evaluation were often defined, providing moderate assurance. General health measures were rarely reported. Procedures for outcome evaluation and subject selection were often biased and unsatisfactorily described. The CMS for the articles was 66, testifying to this study a good quality of the methodologies for the articles included. The CMS is reported in Fig. 2 (Table 1).

**Table 1:** Coleman Methodology Scores for the included articles (mean ± standard deviation)

Endpoint		Value
Part A: only 1 score to be given for each of the 7 sections		
	1. Study size: number of patients	4.3 ± 2.6
	2. Mean follow-up	5.2 ± 2.5
	3. Surgical approach	5.4 ± 4.5
	4. Type of study	6.0 ± 5.6
	5. Description of diagnosis	4.5 ± 1.6
	6. Descriptions of surgical technique	5.1 ± 2.8
	7. Description of postoperative rehabilitation	3.9 ± 2.1
Part B: scores may be given for each option in each of the 3 sections if applicable		
	1. Outcome criteria	2.6 ± 0.6
	2. Procedure of assessing outcomes	3.7 ± 0.9
	3. Description of the subject selection process	4.4 ± 1.6
Total		66.3 ± 7.7

### Study characteristics and results of individual studies

Data from 1848 patients were retrieved. The mean follow-up was 36.8 (9–120) months. The mean age of the patients was 25.8 ± 7.9 years. 28% (521 of 1848 patients) were women. Generalities of the included studies are shown in Table 2.

**Table 2:** Eneralities of the included studies (CMS: Coleman Methodology Score)

Author, year	Journal	Design	CMS	Technique	Follow-up (*months*)	Patients (*n*)	Mean age	Women (*%*)
Ahn et al., 2013 [37]	*Knee Surg Sports Traumatol Arthrosc,*	Prospective	57	All-inside	48.0	13	20.0	46
Barber et al., 2006 [38]	*Arthroscopy*	Retrospective	61	Inside-out	38.6	41	29.8	29
Bryant et al., 2007 [39]	*Am J Sport*	Prospective	59	Inside-out	28.0	49	25.7	41
Chiang et al., 2011 [40]	*Chang Gung Med J*	Prospective	71	All-inside	36.0	31	30.7	39
				All-inside		51	25.1	35
Choi et al., 2009 [41]	*Am J Sport*	Prospective	48	All-inside	35.7	14	28.6	14
				Inside-out		34	27.7	6
Chou et al., 2015 [42]	*Orthop Traumatol Surg Res*	Retrospective	53	All-inside	25.0	24	27.0	25
Diaz- Alvarez et al., 2015 [43]	*Knee Surg Sports Traumatol Arthrosc,*	Prospective	62	All-inside	72.0	29	29.0	0
Gallacher et al., 2010 [44]	*Knee*	Retrospective	50	All-inside	48.0	87	26.0	16
Hagino et al., 2014 [45]	*Eur J Orthop Surg Traumatol*	Prospective	67	All-inside	19.0	57	23.6	53
Haklar et al., 2008 [46]	*Knee*	Prospective	64	Inside-out	31.0	5	28.6	0
Hetsroni et al., 2011 [47]	*Arthroscopy*	Retrospective	60	All-inside	24.0	6		
Hirtler et al., 2015 [48]	*Int Orthop*	Retrospective	55	All-inside	9.0	37	24.2	68
Järvelä et al., 2010 [49]	*Am J Sport Med*	Prospective	83	All-inside	27.0	21	30.0	19
				All-inside	26.0	21	32.0	43
Kamimura et al., 2014 [50]	*Orthop J Sports Med*	Prospective	69	All-inside	12.0	4	52.8	25
				All-inside	12.0	3	32.0	67
Kise et al., 2015 [51]	*Knee Surg Sports Traumatol Arthrosc*	Prospective	73	All-inside	24.0	21	26.9	57
				All-inside	24.0	25	25.5	32
Kotosolov et al., 2006 [52]	*Arthroscopy*	Prospective	71	All-inside	18.0	58	32.6	
Krych et al., 2008 [53]	*Am J Sport Med*	Retrospective	68	Inside-out	24.0	44	15.8	14
Aaron J Krych et al., 2010 [54]	*Am J Sport Med*	Retrospective	77	Inside-out	69.6	99	16.0	57
Logan et al., 2009 [55]	*Am J Sport Med*	Retrospective	71	Inside-out	60.0	42	23.2	31
Lucas et al., 2015 [56]	*Orthop Traumatol Surg Res*	Retrospective	71	All-inside	22.0	17	14.0	47
Moatshe et al., 2018 [57]	*Am J Sport Med*	Prospective	71	Inside- out	36.0	40	32.9	38
				Inside- out	36.0	45		0
Nakayama et al., 2017 [58]	*Asia Pac J Sports Med Arthrosc Rehabil Technol*	Retrospective	63	Inside-out	19.8	46	22.9	26
Noyes et al., 2002 [59]	*Am J Sport Med*	Prospective	67	Inside-out	24.0	58	16.0	45
Noyes et al., 2011 [60]	*Am J Sport Med*	Prospective	70	Inside-out	120.0	31	15.4	45
Olsen et al., 1998 [61]	*Acta Orthop Scand*	Retrospective	62	Inside-out		29	28.0	0
Papachristou et al., 2003 [62]	*Acta Orthop Belg*	Prospective	68	Inside-out	36.0	45	21.8	18
Perdue et al. 1996 [63]	*Arthroscopy*	Retrospective	61	Inside-out	26.9	63	26.9	25
Popescu et al., 2013 [64]	*Knee Surg Sports Traumatol Arthrosc,*	Prospective	64	All-inside	18.5	28	33.0	21
Pujol et al., 2012 [65]	*Knee Surg Sports Traumatol Arthrosc,*	Retrospective	53	All-inside	24.0	19	25.0	32
Raza et al., 2011 [66]	*J Pak Med Ass*	Retrospective	64	Inside-out	17.0	14	41.2	64
Reja et al., 2014 [67]	*Arthroscopy*	Retrospective	61	Inside-out	48.0	24	22.8	21
Schmitt et al., 2016 [68]	*Orthop Traumatol Surg Res*	Prospective	69	All-inside	72.0	19	14.8	37
Spindler et al., 2003 [69]	*Am J Sport Med*	Prospective	68	Inside-out	68.0	47	24.4	44
				All-inside	27.0	98	23.2	48
Steadman et al., 2015 [70]	*Am J Sport Med*	Prospective	75	Inside-out	120.0	136	27.0	32
Thomas Stein et al., 2010 [71]	*Am J Sport Med*	Prospective	67	Inside-out	36.0	42	31.3	38
Tengrootenhuysen et al., 2011 [72]	*Knee Surg Sports Traumatol Arthrosc,*	Retrospective	76	Inside-out	70.0	119	23.0	35
Tiftikçi et al., 2017 [73]	*J Orthop Surg Res,*	Retrospective	63	All-inside	29.0	27	40.5	41
Tucciarone et al., 2012 [74]	*Arch Orthop Trauma Surg*	Prospective	67	All-inside	24.0	40	23.0	13
Vanderhaave et al., 2011 [75]	*J Pediatr Orthop*	Prospective	48	Inside-out	27.0	45	13.2	31

### Results of syntheses

Comparability was found in mean age, rate of women, time span from injury to surgery, Tegner scale, Lysholm and IKDC scores (*P* > 0.05). Comparability of the demographic baseline is shown in detail in Table 3

**Table 3:** Demographic of the included studies

Endpoint	All inside	Inside out	MD	P
Mean age	27.8 ± 8.0	24.5 ± 6.8	− 3.35	0.07
Women (%)	35.3 ± 17.8	29.1 ± 17.7	− 6.29	0.1
Time from surgery to failure (months)	21.3 ± 11.4	23.3 ± 1.7	2.01	0.4
Tegner Activity Scale	4.7 ± 2.0	4.1 ± 1.7	− 0.60	0.3
Lysholm score	60.0 ± 9.3	52.3 ± 6.4	− 7.67	0.08
IKDC Score	40.9 ± 17.5	44.4 ± 22.7	3.48	0.4

No difference was found in PROMs: Tegner Activity Scale (*P* = 0.4), Lysholm score (*P* = 0.2), IKDC score (*P* = 0.4) among patients undergoing meniscal repair with all inside or inside-out technique. These results are shown in greater detail in Table 4.

**Table 4:** Results of PROMs (MD: mean difference)

Endpoint	All inside	Inside out	MD	*P*
Tegner Activity Scale	6.1 ± 0.9	6.0 ± 0.9	− 0.08	0.4
Lysholm score	90.7 ± 5.6	89.3 ± 5.1	− 1.41	0.2
IKDC score	85.5 ± 4.8	85.9 ± 4.4	0.43	0.4

The all-inside repair resulted in a greater rate of re-injury (OR 2.7; 95% CI 1.29–5.74; *P* = 0.009), but also a greater rate of return to play at pre-injury level (OR 2.2; 95% CI 1.48–3.22; *P* = 0.0001). No difference was found in failures (*P* = 0.7), chronic pain (*P* = 0.05), and reoperation (*P* = 0.1) between the two techniques. No difference was found in the rate of return to play (*P* = 0.5), and to daily activities (*P* = 0.1) between the two techniques. These results are shown in greater detail in Table 5.

**Table 5:** Results of binary comparisons (MD: mean difference, CI: confidence interval)

Endpoint	All-Inside	Inside-Out	OR	95% CI	*P*
Failures	18% (78 of 444)	17% (167 of 1001)	1.1	0.79–1.43	0.7
Re-Injury	26% (12 of 46)	11% (35 of 305)	2.7	1.29–5.76	0.008
Chronic Pain	10% (19 of 189)	5% (9 of 192)	2.3	1.00–5.16	0.05
Reoperation	18% (60 of 329)	14% (91 of 643)	1.4	0.94–1.93	0.1
Return to Play	84% (16 of 19)	78% (155 of 199)	0.7	0.18–2.37	0.5
Return to daily activities	85% (163 of 191)	79% (151 of 191)	1.5	0.90–2.62	0.1
Return to play at pre-injury level	75% (132 of 175)	58% (289 of 495)	2.2	1.48–3.22	0.0001

These results are based on the real number of events and observation reported by each single study

## Discussion

According to the main findings of the present meta-analysis, arthroscopic all-inside meniscal repair demonstrated a greater rate of re-injury and return to play at the pre-injury level compared to the inside-out meniscal repair technique. Arthroscopic all-inside meniscal repair may be of special interest in patients with a particular interest in a fast return to sport, while, for less demanding patients, the inside-out suture technique may be recommended.

Many surgeons advocate the inside-out technique to repair the meniscus, as it allows a more secure and perpendicular suture at the side of the lesion [76, 77]. Moreover, the inside-out meniscal repair is versatile and can be performed in all types of meniscal tears of the posterior horn or body [78–80]. However, during arthroscopy accessory posteromedial or posterolateral skin incisions are required for the execution of the suture [81, 82]. Using the inside-out technique, sutures are introduced intra-articularly and are knotted on the capsule. In recent times, the all-inside technique has become increasingly popular [83, 84]. Devices have been introduced to allow all-inside meniscal suture [2, 85]. These devices consist of an anchoring component to the meniscal wall with a sliding and self-locking knot, which allows compression of the injured meniscal fragments [73]. These tools make the meniscal suture surgical technique much easier and simpler, reducing surgical time and the risk of neurovascular complications. Regardless of the repair technique, the present study demonstrated an improvement in PROMs in patients undergoing meniscal sutures. However, whether inside-out performs better than the all-inside meniscal repair technique is debated, and no consensus has been reached. According to our findings, patients undergoing all inside meniscal repair demonstrated a greater risk of re-injury but also a greater rate of return to play at the pre-injury level. Previous clinical investigations included in the present study inferred the same conclusions [37, 43, 48, 49, 54, 65, 74, 86]. No further differences in symptoms, failures, and return to normal activities have been evidenced. In the present study, no difference was found in PROMs and rates of surgical failure, chronic pain, and reoperation. No difference was found in the rate of patients unable to return to play and in the rate of return to daily activities. Hence, all inside meniscal repair may be of special interest to patients who desire a fast return to sport, while, for less demanding patients, the inside-out suture technique may be recommended.

We were able to identify only two clinical studies which compared inside-out versus all inside techniques for meniscal repair [41, 69]. Choi et al. [41]conducted a comparative clinical study on 48 consecutive patients who underwent meniscal repairs of longitudinal tears of the posterior horn of the medial meniscus combined with anterior cruciate ligament reconstructions [41]. At approximately three years of follow-up, no difference was found in ROM and meniscal healing at MRI [41]. Lachman test, KT-1000 arthrometer side-to-side differences, Lysholm scores, and Tegner activity scales were also similar between the two groups [41]. One patient in the inside-out group required manipulation, and two patients had limited ROM [41]. Two transient saphenous nerve injuries were observed in the inside-out group [41]. Spindler et al. [69] comparatively assessed 125 arthroscopic meniscal repairs [69]. The rate of failures (meniscal re-operation) was similar between the groups [69]. Both Kaplan–Meier curves and the Cox proportional hazards model evidenced no difference in time to reoperation between techniques [69].

This study has some limitations. The sample size and length of the follow-up were not adequate in some studies. Moreover, 43% (17 of 40) of the included investigations were retrospective, which increases the risk of selection bias in the present study. Only two comparative clinical trials were included, and all other studies were observational studies. The studies which reported the outcomes of meniscal repair were included irrespective of the type and location of the lesion. However; most studies did not report information on these endpoints or did not conduct the analyses of the patients separately. No information was given in relation to the previous conservative management, for example, platelet-rich plasma injection. Rehabilitation protocols were often biased and general health measures were not reported. Procedures for outcome evaluation and subject selection were often biased and unsatisfactorily described. Most authors did not report information on the injury onset (acute or chronic); therefore, no further subgroup analyses were possible. Many authors performed other procedures (e.g. anterior cruciate ligament) in association with the meniscal repair; therefore, results might be not fully generalizable. Further high-quality comparative studies are required to validate the results of the present study in a clinical setting.

## Conclusion

Arthroscopic all-inside meniscal repair demonstrated a greater rate of re-injury and return to play at the pre-injury level compared to the inside-out meniscal repair technique. Arthroscopic all-inside meniscal repair may be of special interest in patients who wish for a fast return to sport, while, for less demanding patients, the inside-out suture technique may be recommended. High-quality comparative trials are required to validate these results in a clinical setting and to evaluate the potential of these techniques according to the type and place of the lesion.

## Data Availability

The datasets generated during and/or analysed during the current study are available throughout the manuscript.

## Abbreviations

PROMs: Patient-reported outcome measures
MD: Mean difference
OR: Odd ratio
CI: Confidence interval
IKDC: International knee documentation committee
CMS: Coleman methodology score
